# WY7 is a newly identified promoter from the rubber powdery mildew pathogen that regulates exogenous gene expression in both monocots and dicots

**DOI:** 10.1371/journal.pone.0233911

**Published:** 2020-06-01

**Authors:** Yi Wang, Chen Wang, Mamy Jayne Nelly Rajaofera, Li Zhu, Wenbo Liu, Fucong Zheng, Weiguo Miao

**Affiliations:** 1 Key Laboratory of Green Prevention and Control of Tropical Plant Diseases and Pests, Hainan University, Ministry of Education, Haikou, China; 2 College of Plant Protection, Hainan University, Haikou, China; Fujian Agriculture and Forestry University, CHINA

## Abstract

Promoters are very important for transcriptional regulation and gene expression, and have become invaluable tools for genetic engineering. Owing to the characteristics of obligate biotrophs, molecular research into obligate biotrophic fungi is seriously lagging behind, and very few of their endogenous promoters have been developed. In this study, a WY7 fragment was predicted in the genome of *Oidium heveae* Steinmann using PromoterScan. Its promoter function was verified with transient transformations (*Agrobacterium tumefaciens*-mediated transformation, ATMT) in *Nicotiana tabacum* cv. Xanthi nc. The analysis of the transcription range showed that WY7 could regulate *GUS* expression in both monocots (*Zea mays* Linn and *Oryza sativa* L. spp. Japonica cv. Nipponbare) and dicots (*N*. *tabacum* and *Hylocereus undulates* Britt). The results of the quantitative detection showed that the *GUS* transient expression levels when regulated by WY7 was more than 11.7 times that of the CaMV 35S promoter in dicots (*N*. *tabacum*) and 5.13 times that of the ACT1 promoter in monocots (*O*. *sativa*). GUS staining was not detected in the T1 generation of the WY7-*GUS* transgenic *N*. *tabacum*. This showed that WY7 is an inducible promoter. The cis elements of WY7 were predicted using PlantCARE, and further experiments indicated that WY7 was a low temperature- and salt-inducible promoter. Soluble proteins produced by WY7-*hpa1Xoo* transgenic tobacco elicited hypersensitive responses (HR) in *N*. *tabacum* leaves. *N*. *tabacum* transformed with pBI121-WY7-*hpa1Xoo* exhibited enhanced resistance to the tobacco mosaic virus (TMV). The WY7 promoter has a lot of potential as a tool for plant genetic engineering. Further in-depth studies will help to better understand the transcriptional regulation mechanisms of *O*. *heveae*.

## Introduction

Gene expression is largely regulated by promoters, making them important tools in genetic engineering. Nowadays, research on gene expression is deeper and more meticulous than ever. Constitutive expression, inducible expression, time-specific expression, and space-specific expression of exogenous genes in transgenic hosts are all foci of gene expression research. Furthering these research directions urgently requires more varieties promoters to be developed and applied, especially with regard to genetically modified crops required for human food-security and well-being. Particularly, the endogenous promoters of filamentous fungi have been relatively underreported. One example is the constitutive *gpdA* promoter in *Aspergillus nidulans* [1]. Another is the inducible *cbh1* promoter that is induced by lactose, cellulose, and cellobiose, which is mainly used to express heterologous genes in *Trichoderma reesei* [2]. The ethanol-inducible promoter alcA in *Aspergillus nidulans* has been used in a wide range of studies on objective genes [3].

Obligate biotrophic fungi cannot be cultured *in vitro*, limiting the molecular research strategies that can be utilized; this may explain why molecular research into this class of organisms is severely lacking. Consequently, there are very few reported promoters that have been derived from obligate biotrophic fungi.

Harpins are a class of proteins produced by gram-negative phytopathogenic bacteria and secreted by type III protein secretion systems [4]. They are mostly water-soluble acidic proteins, rich in glycine and lacking cysteine; they are generally thermostable and sensitive to proteinase K and ultraviolet light [5]. HarpinXoo, as a protein elicitor, can induce hypersensitive responses (HR), and is encoded by the *hrp* gene cluster gene *Hpa1Xoo* of *Xanthomonas oryzae* pv. A specific feature of harpinXoo is that it has cysteine, which is not found in other harpins [6]. The exogenous application of harpins can induce resistance in plants to fungal, bacterial, and viral diseases. Meng *et al*. reported that *Hpa1Xoo* transgenic tobacco had increased tobacco mosaic virus (TMV) resistance [7]. Dong *et al*. demonstrated that *Arabidopsis* resistance to *Pseudomonas syringae* pv. *tomato* and *Peronospora parasitica* was enhanced by the induction of harpin_Ea_ [8].

In this study, we aimed to identify and develop an endogenous promoter of obligate biotrophic fungus *Oidium heveae*, the pathogen of rubber powdery mildew which is one of the most economically damaging diseases of rubber trees (*Hevea brasiliensis*). Through the prediction and analysis of the genome of *O*. *heveae*, we identified a new promoter called WY7. We used WY7 to regulate the transient expression of the *GUS* gene in tobacco and verified its promoter function. In studying the manner in which WY7 regulated the expression range and level of exogenous genes in plants, we discovered that it had the potential to be an excellent promoter for use in genetic engineering. Our results also laid the foundation for follow-up studies on the function of the genes regulated by WY7 in *O*. *heveae* and the pathogenic mechanisms of *O*. *heveae*.

## Materials and methods

### Pathogen strain and plant materials

*O*. *heveae* Steinmann strain HO-73 was inoculated on the medium-susceptible *Hevea brasiliensis* Reyan 7-33-97 at 25°C and in 88% relative humidity. *Nicotiana tabacum* cv. Xanthi nc, *Hylocereus undulates* Britt, *Oryza sativa* L. spp. Japonica cv. Nipponbare and *Zea mays* Linn were cultured in the growth chamber (25°C) with a photoperiod of 16/8 h (light/ dark).

### Promoter prediction

The genome sequence of *O*. *heveae was* analyzed using PromoterScan (http://www-bimas.cit.nih.gov/molbio/proscan/) to predict the promoters [9–12].

### WY7 promoter function verification

#### Construction of the expression vector

Specific primers for WY7 (WY7F and WY7R) were designed using Primer Premier 5.0 (Premier Biosoft International, CA, USA), and BamHI and HindIII restriction sites were introduced. The WY7 fragment was amplified from the genome of *O*. *heveae*. After TA cloning (Promega, pGEM®-T Easy Vector Systems), it was introduced into the pBI121 vector to replace the 35S promoter for *GUS* gene regulation. Finally, the recombinant vector pBI121-WY7 was constructed.

#### Tri-parental mating

The pBI121-WY7 was introduced into *Agrobacterium tumefaciens* LBA4404 to obtain an *Agrobacterium* strain of WY7 using the tri-parental mating method described by Wei *et al*. [13]. All *Agrobacterium* strains were constructed by tri-parental mating with *E*. *coli* DH5α as the donor strain carrying the recombinant plasmids constructed to contain the WY7 and *E*. *coli* HB101 (pRK2013) as the helper strain. The transconjugants were obtained by spreading the mixture on *Agrobacterium tumefaciens* LBA4404 with kanamycin (50 μg mL^−1^) and rifampicin (100 μg mL^−1^).

#### Transient expression of the *GUS* gene

WY7 was transient transformed into the *N*. *tabacum* genome by *Agrobacterium tumefaciens*-mediated transformation (ATMT), and GUS staining analysis was then immediately performed [14–16]. *Agrobacterium* strains of pBI121-WY7 were single-spot cultured to an OD_600_ value of 0.6–0.8 in LB medium, and then used to infect tobacco leaf discs. The discs were then co-cultured in the dark for 2 d in MS medium containing 6-BA (1.0 mg L^-1^) and NAA (0.1 mg L^-1^). After the co-cultivation, the leaf discs were stained.

### Expression range regulated by the WY7

In order to explore its expression range, WY7 was transiently expressed by ATMT both in dicots (*N*. *tabacum*, *H*. *undulates)* and monocots (*O*. *sativa* and *Z*. *mays)*; then, the GUS staining was analyzed.

#### *GUS* transient expression levels regulated by WY7

After transient transformations, the RNA of *N*. *tabacum* and *O*. *sativa* were extracted and reverse transcribed to obtain cDNA; then, the transient expression levels were determined by quantitative qRT-PCR. qRT-PCR was performed using a QIAGEN Rotor-Gene Q MDx RealTime PCR system with the following PCR conditions: 94°C for 30 s; then 45 cycles of 94°C for 12 s, 58°C for 30 s, and 72°C for 30 s; and finally, 72°C for 10 min. Actin was used to normalize the mRNA levels, using the primers ActinF (5′-TCCTCATGCAATTCTTCGGT-3′) and ActinR (5′-TTCCAACAAGTGATGGCTGG-3′). The *GUS* primers used were GUSF (5′-GTCGCGCAAGACTGTAACCA-3′) and GUSR (5′-TGGTTAATCAGGAACTGTTG-3′). The relative expression levels of the *GUS* gene were calculated using the 2^-ΔΔCt^ method [17, 18]. Data were obtained from three sets of experiments and analyzed using SPSS version 16.0 software (SPSS Inc., Chicago, IL, USA).

### The analysis of WY7 promoter type

#### WY7-*GUS* transgenic *N*. *tabacum*

The WY7-*GUS* was stably expressed in *N*. *tabacum* by ATMT. After dark co-cultivation of the ATMT-transformed tobacco leaf discs, they were transferred to MS solid medium plates containing kanamycin (10 μg mL^-1^), 6-BA (1.0 mg L^-1^), and NAA (0.1 mg L^-1^) and then cultured at 26°C with a photoperiod of 16/8 h (light/ dark). Approximately 2 weeks later, adventitious buds appeared; once they were 1 to 2 cm tall, they were cut with a sterilized scalpel and inoculated into 1/2 MS medium containing kanamycin (10 μg mL^-1^) for rooting. They were then cultured at 26°C for approximately 2 weeks. The tobacco seedlings that showed good rooting were selected, the medium was washed away, and the seedlings were then transplanted into sterilized soil for potting. Seeds were harvested approximately 2 months after potting. Two weeks after sowing the seeds, the T1 generation transgenic tobacco seedlings were obtained. *GUS* expression in all organs and tissues was assessed in the T1 generation.

#### Molecular verification

The genome of the T1 generation was extracted. The WY7 was verified by PCR and Southern blot, using the pBI121 transgenic tobacco as the positive control and the *N*. *tabacum* wild type as the negative control. In PCR-Southern blot hybridization, genomic DNA of the WY7-*GUS* transgenic *N*. *tabacum* was PCR amplified using primers WY7F/WY7R and then blotted to nylon membranes. Replicates of blots were hybridized with a digoxigenin-labeled WY7 probe (PCR amplified from *O*. *heveae*). The specific steps of the Southern blot were in accordance with the manufacturer’s guidelines (Roche, DIG-High Prime DNA Labeling and Detection Starter Kit I).

#### Promoter type analysis

*GUS* expression was assessed in all the organs and tissues of the T1 generation using GUS staining. A positive result of some or all of the tissues/organs staining blue would indicate that the WY7 promoter is a constitutive or tissue/organ-specific promoter. A negative result would indicate that WY7 is an inducible promoter. In the case of a negative result, the cis elements of the WY7 sequence would be analyzed using PlantCARE (http://bioinformatics.psb.ugent.be/webtools/plantcare/html/), and the corresponding verification experiments, according to the type of cis-acting element, would be performed.

#### Induction type analysis of WY7

The T1 generation WY7-GUS transgenic tobacco seedlings were induced under several experimental conditions, with three replicates for each set. The salt induction was carried out with 300 and 500 mM NaCl. The seedlings were soaked in a NaCl solution, and the results were observed after 12, 24, and 48 h. This methodology was a slight modification of that reported by Divya *et al*. [19]. The low-temperature induction methodology followed that described by Thomashow *et al*. [20], with slight modifications. The seedlings were induced at 4°C under the following induction time gradient: 0, 4, 8, 12, 24, and 48 h. The dark and continuous light induction methodologies were based on those described by Qu *et al*. [21] with slight modifications. Seedlings were induced in dark and continuous light conditions for 48 and 72 h, respectively. The drought induction followed the PEG method described by Xu *et al*., with minor modifications [22]. The seedlings were induced using 30% PEG6000, under the following induction time gradient: 0, 2, 4, 6, 12, 24, 48 and 72 h. After each induction, GUS staining was conducted following the methodology reported by Kausch *et al*. [23].

### *Hpa1Xoo* expression regulated by WY7 in *N*. *tabacum*

#### Generation of pBI121-WY7-*hpa1Xoo* transgenic plants

The recombinant vector pBI121-WY7 was digested using SacI and BamHI, followed by the removal of the *GUS* gene and the introduction of *Hpa1Xoo*, which had been amplified from *Xanthomonas oryzae* pv. oryzae. The recombinant vector pBI121-WY7-*hpa1Xoo* was then constructed. Tissue cultures and plant regenerations were performed as described in previous sections (WY7-GUS transgenic *N*. *tabacum*). The transformations were verified using PCR and Southern blot.

#### Determination of the phenotype

The expression of WY7-*hpa1Xoo* in the transgenic tobacco was compared with that in pBI121-*hpa1Xoo* transgenic tobacco, pBI121 transgenic *N*. *tabacum*, and *N*. *tabacum* wild type by evaluating phenotypic traits such as fresh weight, dry weight, height, and chlorophyll content [24]. All comparisons were carried out with three replicates per set; each set involved ten plants.

#### Protein analysis and micro-HR observation

Soluble proteins were isolated from the leaves of the T1 generation, following the methodology described previously by Bollag *et al*., with some modifications [25]. The isolated proteins were heated in a boiling-water bath for 10 min; they were then tested for bioactivity and resolved using sodium dodecyl sulfate-polyacrylamide gel electrophoresis (SDS-PAGE). The untreated proteins were used as control. They were evaluated in comparison with the hpa1Xoo proteins from the *E*. *coli* strain BL21/pGEX-*hpa1Xoo*. HR was observed in the tobacco leaves infiltrated with 100 μg mL^−1^ of the protein preparations, indicating that this concentration was sufficient for eliciting a response.

#### Disease scoring

The T1 generation plants were inoculated with TMV (100 μL; 18 μg mL^−1^ of solution) by rub inoculation 40 days after transplantation following a previously described methodology [26] and were then grown in a growth chamber (20–30°C, 16/8 h light/dark). The disease symptoms (mosaic, leaf deformation, and stunting) were assessed after 7 days, following the methodology described by Peng *et al*. [27]. Assays were conducted three times, each involving ten plants. For all quantitative determinations, the data were analyzed by Duncan’s multiple range test at *P* ≤ 0.01. For differences in disease severity, the T1 generation of WY7-*hpa1Xoo* transgenic tobacco and the T1 generation of 35S-*hpa1Xoo* transgenic tobacco were all compared against *N*. *tabacum* wild type.

## Results

### Prediction of the endogenous promoter of *O*. *heveae*

Multiple endogenous promoters were predicted in *O*. *heveae* using PromoterScan (Version 1.7), and one of them was named WY7 (Table 1). No homologous sequence aligning with WY7 was found in the National Center for Biotechnology Information (NCBI) database or in the eukaryotic promoter database (EPD). This confirms that WY7 is a new promoter; consequently, we have applied for a GenBank accession number (MN889519).

**Table 1:** Prediction of the WY7 sequence in *O*. *heveae* genome using PromoterScan. Promoter region predicted on forward strand in 39676 to 39926. Promoter Score: 56.35 (Promoter Cutoff = 53.000000). TATA found at 39898, Est.TSS = 39928.

Significant signals	TFD #	Strand	Location
IgNF-A	S00830	+	39712
GATA-1	S00486	+	39738
CTF	S00780	-	39743
T-Ag	S00974	+	39769
CTF	S00780	+	39867
TFIID	S01540	+	39885
EIIF	S00659	+	39924

TATA, TATA box; TSS, transcription start site; significant signals, transcription factor binding site; TFD, transcription factor database.

### Functional verification of the WY7 promoter

**Fig 1:**
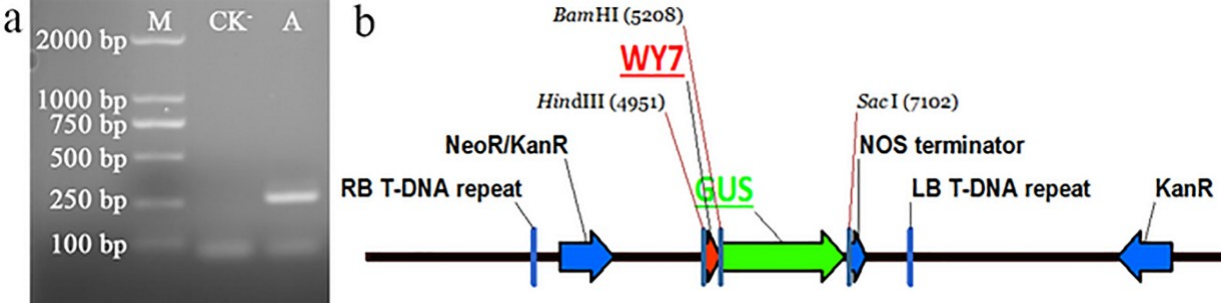
Construction of the plant expression vector of the *O*. *heveae* endogenous suspected promoter WY7. **a)** Amplification of WY7. (M) Marker 2000; (CK^-^) Negative control with ddH_2_O as template; (A) WY7. **b)** Map of recombinant vector pBI121-WY7.

**Fig 2:**
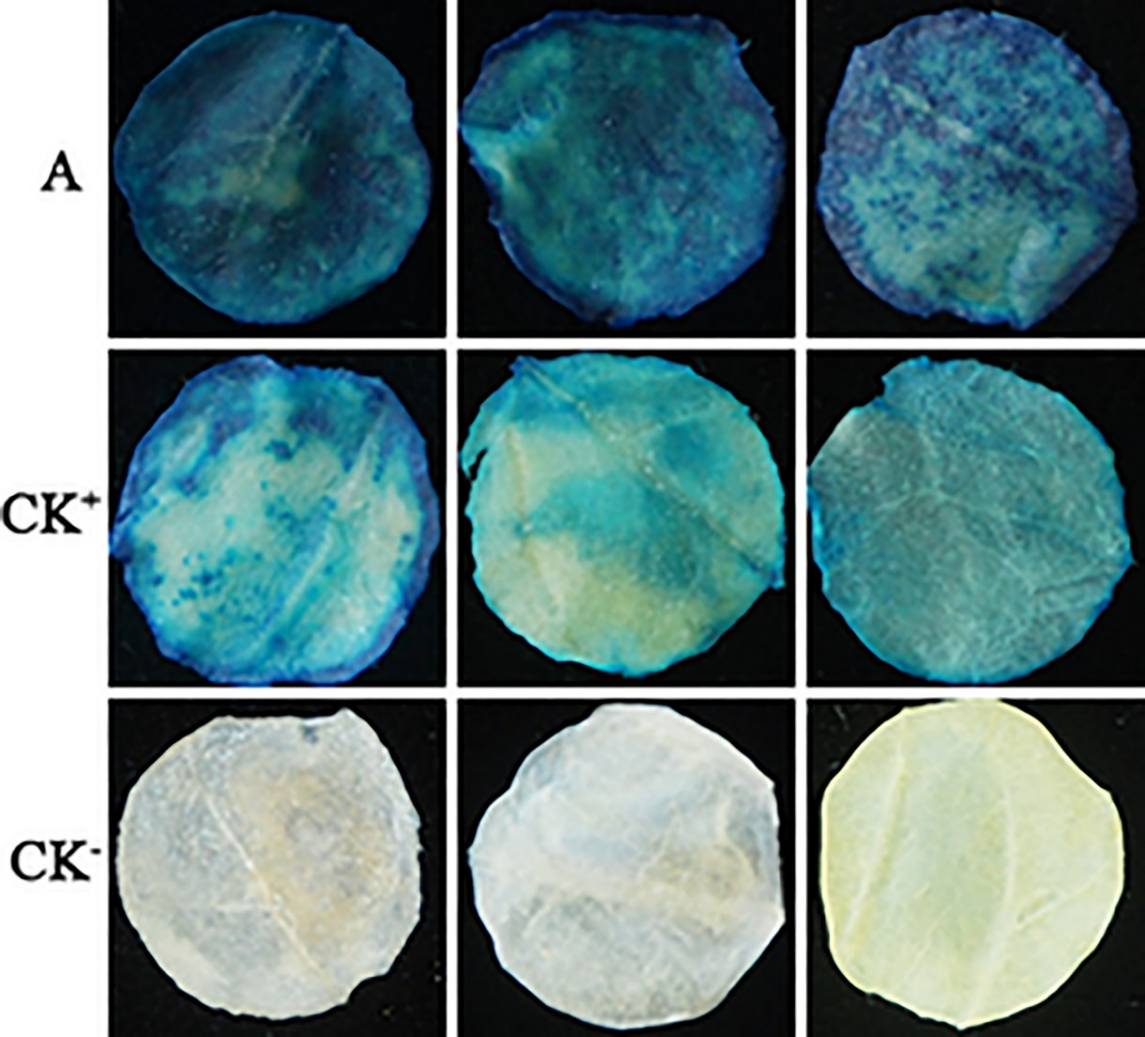
Histochemical staining for GUS activity in transiently transformed leaf discs of *N*. *tabacum*. (A) GUS activity in the leaf discs regulated by WY7; (CK^+^), positive control, GUS activity in the leaf discs regulated by the 35S promoter; (CK^-^), negative control, leaf discs of *N*. *tabacum* wild type.

The promoter fragment of WY7 was amplified using the genomic DNA of *O*. *heveae* strain HO-73 as a template, with the primers WY7F (5′-CCCAAGCTTTTGGAGGTTTCTACAGCCTT-3′) and WY7R (5′-CGGGATCCCGCTTGTCTGAAGTTTTTGC-3′). The electrophoresis results are shown in Fig 1A, and a bright band of ~ 250 bp, which is consistent with the WY7 sequence size predicted by PromoterScan, can be seen. After the TA cloning, the WY7 was introduced into the pBI121 vector to replace the 35S promoter, and the recombinant expression vector pBI121-WY7 was then constructed (Fig 1B). In the tobacco containing the WY7 promoter, staining for up to 24 h showed that the *GUS* gene was expressed in the leaves (Fig 2). In plants containing the 35S promoter, staining in the leaves was relatively weak. GUS staining was not observed in the leaves of the *N*. *tabacum* wild type. The results showed that WY7 could successfully drive the expression of the *GUS* gene, which indicated that it has promoter functions.

### Expression range regulated by WY7

**Fig 3:**
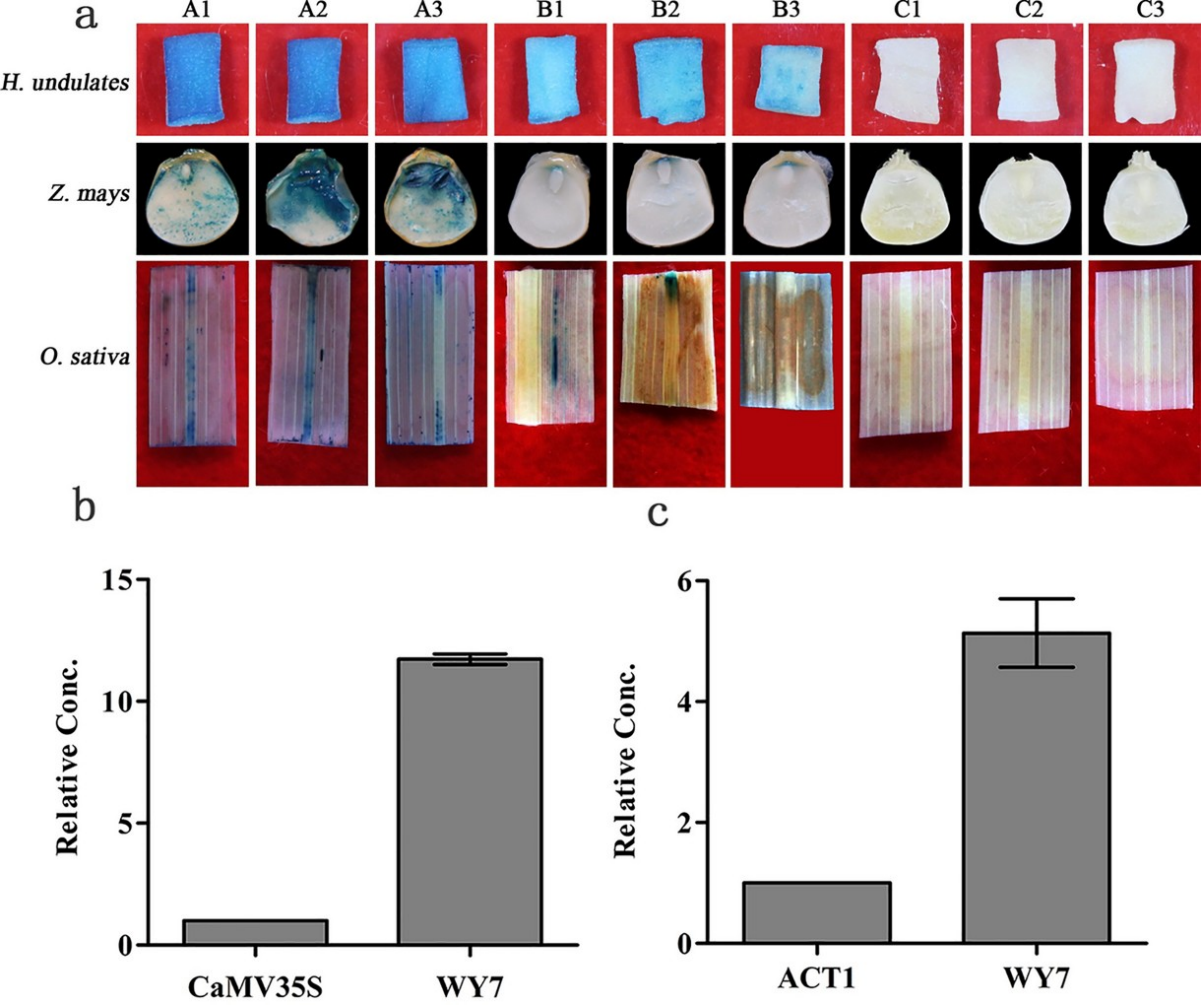
Transient expression of *GUS* regulated by the WY7 promoter in monocots and dicots. **a)** Histochemical staining for GUS activity in transient transformed dicots and monocots. (A1) to (A3) GUS activity in dicots regulated by the WY7 promoter; (B1) to (B3) CK^+^, positive control, GUS activity regulated by the 35S promoter; (C1) to (C3) CK^–^, negative control, GUS activity in wild type. **b)** Transient expression levels in dicots (*N*. *tabacum*); CK^+^, 35S. **c)** Transient expression levels in monocots (*O*. *sativa*); CK^+^, ACT1 promoter.

WY7-*GUS* was transiently expressed in dicots and verified by the GUS staining. The GUS staining in the endosperm of the *H*. *undulates* tender stems (Fig 3A) and tobacco leaves (as described in the preceding functional verification section) was easily observed with the naked eye. In the negative controls, the GUS staining could not be detected. This indicated that the dicots infected with *Agrobacterium* LB4404 containing the pBI121-WY7 recombinant vector could successfully express the exogenous *GUS* gene. The WY7 promoter was active in dicots. In the positive controls, the GUS staining was also observed. The *GUS* gene was expressed in dicots, driven by the 35S promoter.

WY7-*GUS* was also transiently expressed in monocots and verified by GUS staining. The GUS staining in the *Z*. *mays embryos* and *O*. *sativa* leaves (Fig 3A) was easily observed with the naked eye. In the negative controls, the GUS staining could not be detected. This indicated that the monocots infected with *Agrobacterium* LB4404 containing the pBI121-WY7 recombinant vector could successfully express the exogenous *GUS* gene. The WY7 promoter was active in monocots. In the positive controls, the GUS staining was also observed. The *GUS* gene was expressed in monocots driven by the ACT1 promoter.

### Transient expression level regulated by WY7

To further understand the ability of WY7 to regulate the expression of foreign genes, we extracted the RNA of tobacco and *O*. *sativa* leaves after the transient expression. Then, the transient expression level of the *GUS* gene, regulated by WY7, in tobacco (a dicot) was determined by quantitative qRT-PCR and compared with the level recorded when it was regulated by the 35S promoter (the positive control). The transient expression level of the *GUS* gene, regulated by WY7, in *O*. *sativa* (a monocot) was determined by quantitative qRT-PCR and compared with the level recorded when it was regulated by the ACT1 promoter (the positive control). The results showed that the expression level of the *GUS* gene under WY7 regulation in tobacco was 11.7-fold higher than that under the 35S promoter regulation, and the expression level under WY7 regulation in *O*. *sativa* was 5.13-fold higher than that under the ACT1 promoter regulation (Fig 3B and 3C).

### WY7 promoter type

**Fig 4:**
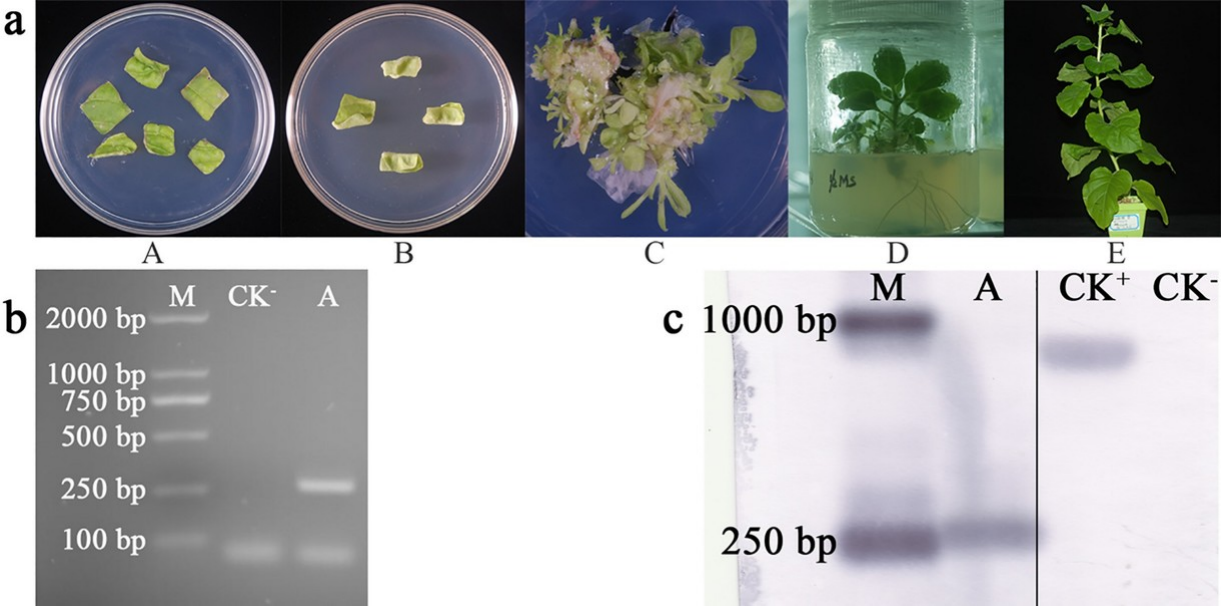
Stable expression of WY7 in tobacco. **a)** Stages of *Agrobacterium*-mediated tobacco transformation. (A) Tobacco leaf discs for co-cultivation with *Agrobacterium* inoculum. (B) Callus of *N*. *tabacum*. (C) Adventitious buds. (D) 14-day-old plantlets that survived on Kanamycin selection media transplanted to a jar. (E) Mature plants transplanted to soil. **b)** Amplification of WY7. (M) Marker 2000; (CK^-^) Negative control with ddH_2_O as template; (A) WY7. **c)** PCR-Southern blot analysis. (M) DNA molecular weight marker (DIG-labeled); (A) WY7; (CK^–^) *N*. *tabacum* wild type (Fragments of the same original image were spliced together to remove irrelevant lanes, complete blotsgels are presented in S1–S5 Figs).

**Fig 5:**
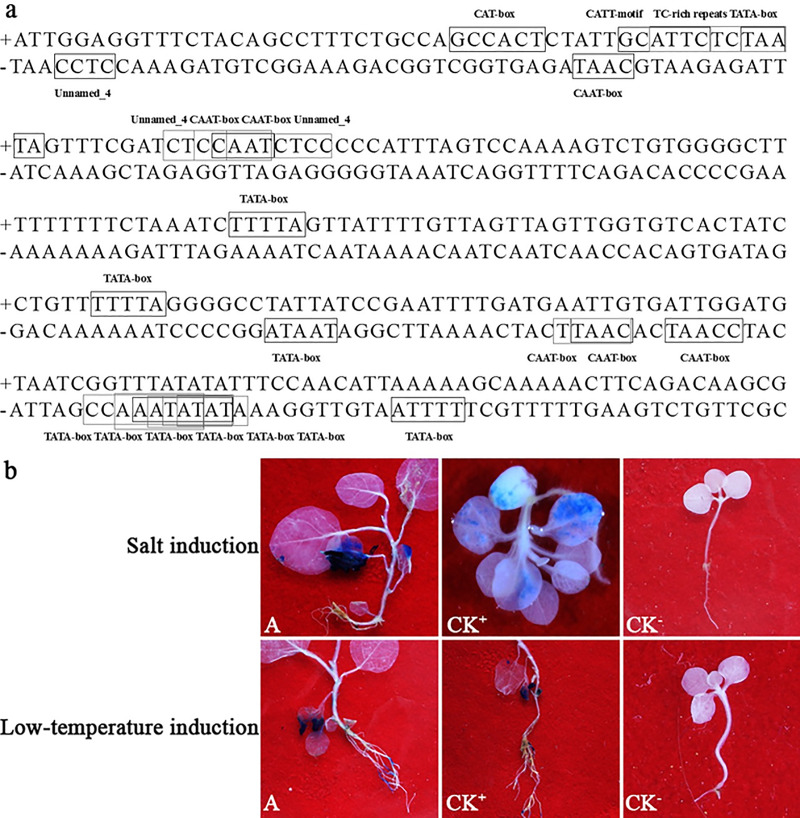
Analysis of WY7 promoter type. **a)** Sequence analysis of the WY7 promoter using PlantCARE. **b)** Histochemical staining for GUS activity in a T1 generation of WY7 transgenic tobacco grown under different conditions. (A) WY7-*GUS* transgenic tobacco; (CK^+^) 35S-*GUS* transgenic tobacco; (CK^-^) *N*. *tabacum* wild type.

WY7 was stably expressed in the T1 generation of the transgenic *N*. *tabacum* (Fig 4A), and the PCR verification showed a characteristic band at ~ 250 bp, which was consistent with the size of the WY7 promoter (Fig 4B). Similarly, the Southern blot results (Fig 4C) for the transgenic tobacco plants detected hybrid bands of ~ 250 bp, which is consistent with the WY7 sequence size predicted by PromoterScan; this indicated that WY7 was successfully integrated. GUS staining was not detected in any of the tissues or organs of the T1 generation. Therefore, we conclude that WY7 is not a constitutive or tissue/organ-specific promoter, but an inducible promoter. In order to determine the WY7 induction type, the cis elements of the WY7 were predicted by PlantCARE, the results of which are shown in Fig 5A and Table 2. In WY7, there was a cis-acting element involved in defense and stress responsiveness, a light responsive element, a cis-acting regulatory element related to the meristem expression, and two unknown cis-acting regulatory elements. Therefore, related inductions, according to the types of its cis-acting elements, were carried out. In the eukaryotic promoter, temperature and salt are common inducing types. As there are three unknown cis-acting regulatory elements in WY7, temperature and salt inductions were carried out. WY7 was found to be a low temperature and salt-inducible promoter (Fig 5B).

**Table 2:** Sequence analysis of the WY7 promoter using PlantCARE.

Cis-acting regulatory elements	Organism^a^	Position^b^	Strand^c^	Sequence	Function
TATA-box	*Glycine max*	47	+	TAATA	Core promoter element around -30 of transcription start
	*Arabidopsis thaliana*	208	-	TATATAA	Core promoter element around -30 of transcription start
	*Arabidopsis thaliana*	205	-	TATATAAATC	Core promoter element around -30 of transcription start
	*Lycopersicon esculentum*	225	-	TTTTA	Core promoter element around -30 of transcription start
	*Lycopersicon esculentum*	155	+	TTTTA	Core promoter element around -30 of transcription start
	*Brassica napus*	210	-	ATATAT	Core promoter element around -30 of transcription start
	*Arabidopsis thaliana*	207	-	TATAAA	Core promoter element around -30 of transcription start
	*Arabidopsis thaliana*	209	-	TATA	Core promoter element around -30 of transcription start
	*Lycopersicon esculentum*	114	+	TTTTA	Core promoter element around -30 of transcription start
	*Arabidopsis thaliana*	211	-	TATA	Core promoter element around -30 of transcription start
	*Glycine max*	166	-	TAATA	Core promoter element around -30 of transcription start
CAAT-box	*Hordeum vulgare*	36	-	CAAT	Common cis-acting element in promoter and enhancer regions
	*Hordeum vulgare*	186	-	CAAT	Common cis-acting element in promoter and enhancer regions
	*Hordeum vulgare*	63	+	CAAT	Common cis-acting element in promoter and enhancer regions
	*Arabidopsis thaliana*	192	-	CCAAT	Common cis-acting element in promoter and enhancer regions
	*Arabidopsis thaliana*	62	+	CCAAT	Common cis-acting element in promoter and enhancer regions
	*Glycine max*	185	-	CCAAT	Common cis-acting element in promoter and enhancer regions
CAT-box	*Arabidopsis thaliana*	28	+	GCCACT	Cis-acting regulatory element related to meristem expression
CATT-motif	*Zea mays*	39	+	GCATTC	Part of a light responsive element
TC-rich repeats	*Nicotiana tabacum*	41	+	ATTCTCTAAC	cis-acting element involved in defense and stress responsiveness
Unnamed_4	*Petroselinum hortense*	3	-	CTCC	
Unnamed_4	*Petroselinum hortense*	67	+	CTCC	
Unnamed_4	*Petroselinum hortense*	60	+	CTCC	

^a^ Organism: organisms with this cis-acting element that have been reported

^b^ Position: the position of this cis-acting element in WY7

^c^ Strand: the presence (+) or absence (-) of this cis-acting element exists in the sense or antisense strand of the *O*. *heveae* genome.

### *Hpa1Xoo* expression regulated by WY7 in *N*. *tabacum*

#### Phenotype determination of pBI121-WY7-*hpa1Xoo* transgenic plants

**Fig 6:**
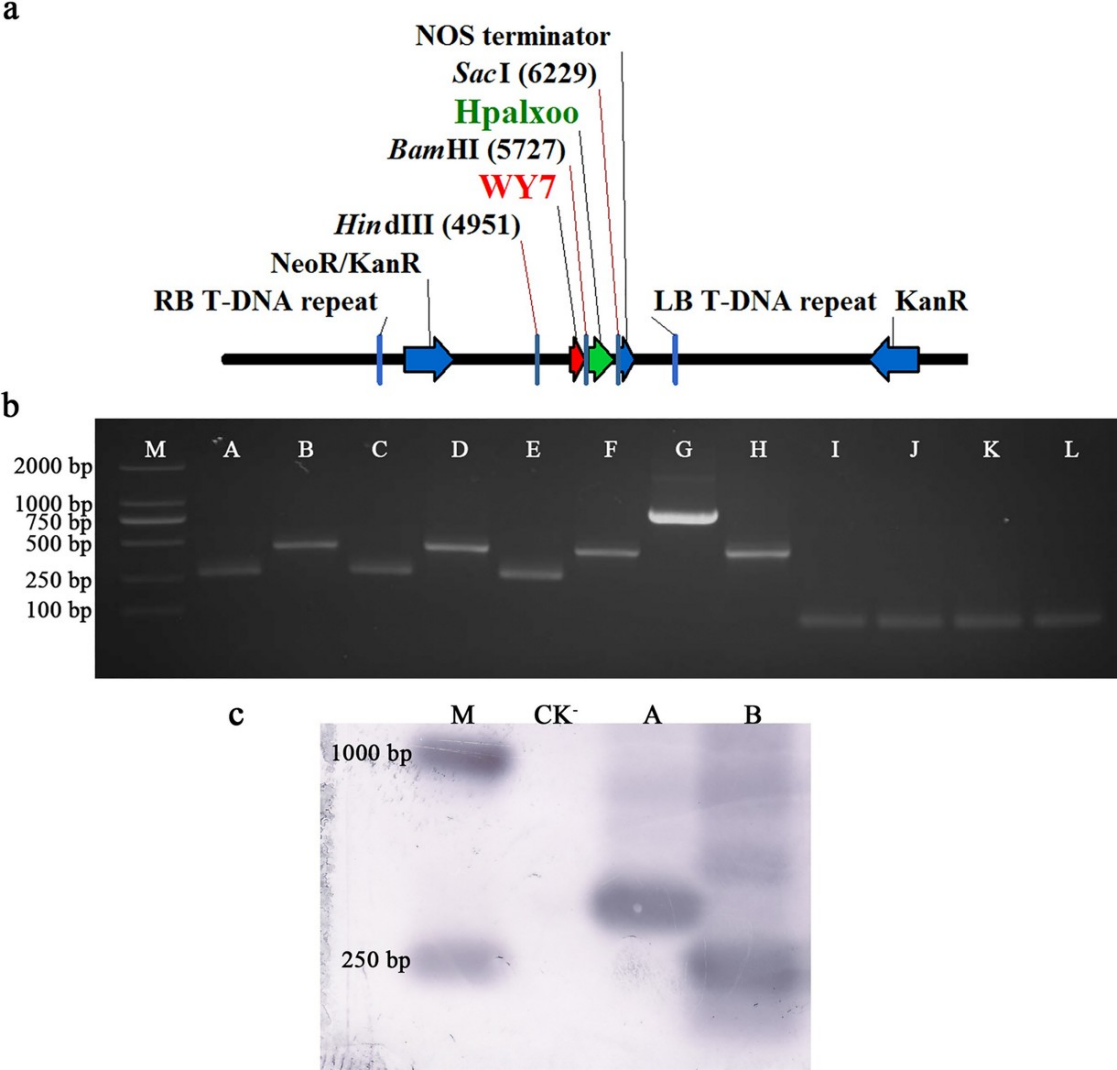
WY7-regulated expression of *Hpa1Xoo* in tobacco. **a)** Map of the recombinant vector pBI121-WY7-*Hpa1Xoo*. **b)** PCR amplification. (M) Marker 2000; (A) WY7-*Hpa1Xoo* transgenic tobacco plants, primers: WY7F/R; (B) WY7-*Hpa1Xoo* transgenic tobacco plants, primers: Hpa1XooF/R; (C) CK^+^, template: *O*. *heveae* wild type, primers: WY7F/R; (D) CK^+^, template: *X*. *oryzae* wild type, primers: Hpa1XooF/R; (E) CK^+^, template: recombinant vector pBI121-WY7-*Hpa1Xoo*, primers: WY7F/R; (F) CK^+^, template: recombinant vector pBI121-WY7-*Hpa1Xoo*, primers: Hpa1XooF/R; (G) CK^+^, template: 35S-*Hpa1Xoo* transgenic tobacco plants, primers: 35SF/R; (H) CK^+^, template: 35S-*Hpa1Xoo* transgenic tobacco plants, primers: Hpa1XooF/R; (I) CK^-^, template: *N*. *tabacum* wild type, primers: WY7F/R; (J) CK^-^, template: *N*. *tabacum* wild type, primers: Hpa1XooF/R; (K) CK^-^, template: ddH_2_O, primers: WY7F/R; (L) CK^-^, template: ddH_2_O, primers: Hpa1XooF/R. **c)** PCR-Southern blot analysis. (M) DNA molecular weight marker (DIG-labeled); (CK^–^) *N*. *tabacum* wild type; (A) WY7; (B) *Hpa1Xoo*.

**Fig 7:**
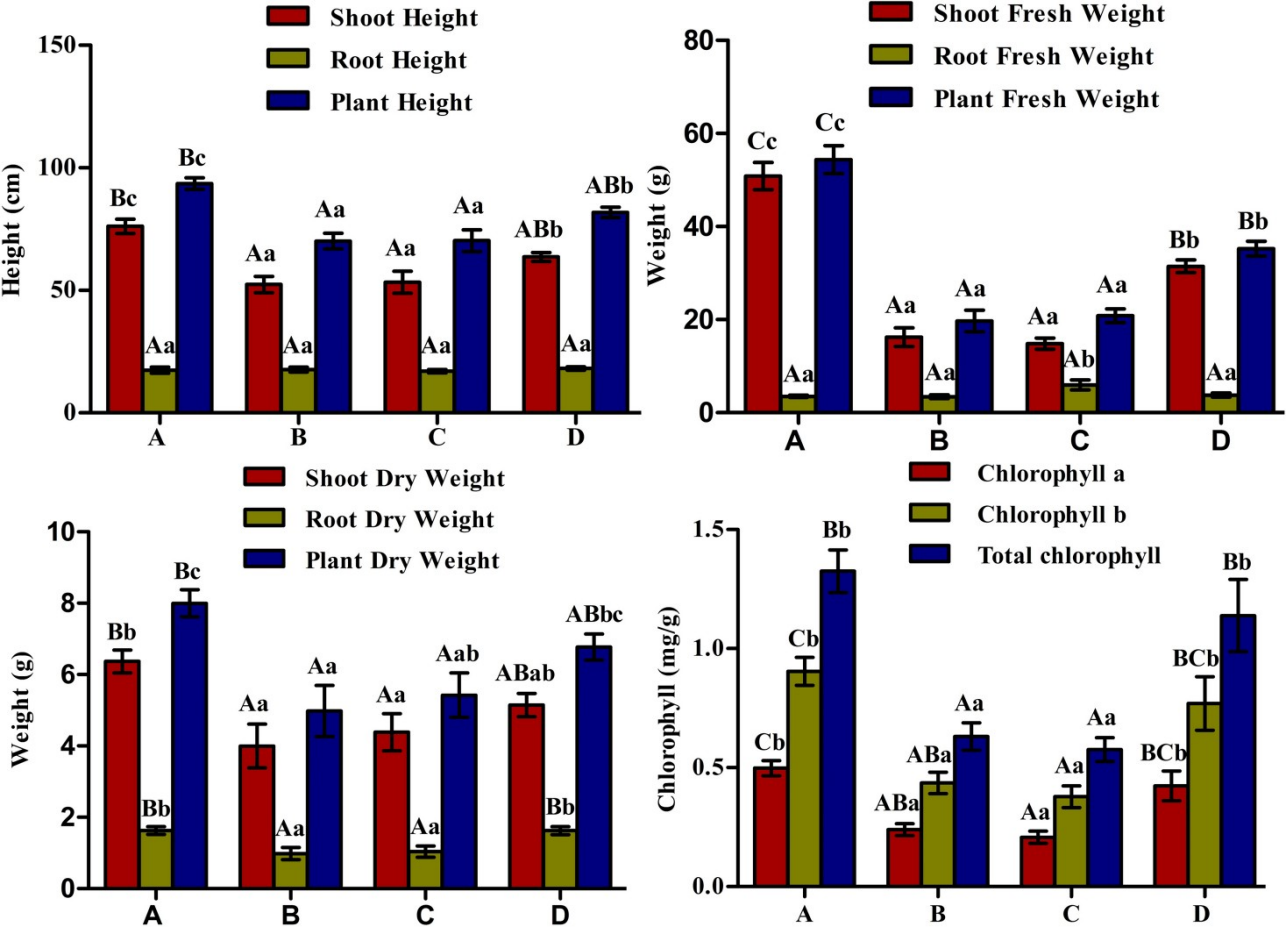
Phenotype determination of pBI121-WY7-Hpa1Xoo transgenic plants. Plant height, plant fresh weight, plant dry weight, and total chlorophyll content of the pBI121-WY7-*Hpa1Xoo* transgenic tobacco were measured. (A) WY7-*Hpa1Xoo* transgenic tobacco; (B) CK^-^, *N*. *tabacum* wild type; (C) CK^-^, pBI121 transgenic tobacco. (D) CK^+^, 35S-*Hpa1Xoo* transgenic tobacco.

The WY7-*hpa1Xoo* was transformed into *N*. *tabacum* by ATMT. *Hpa1Xoo* was stably expressed in the T1 generation (Fig 6A). Positive results were obtained from the PCR verification, and the amplified product exhibited a length of around 420 bp (Fig 6B). The Southern blot results showed that the transgenic plants had hybrid bands that were ~ 250 and 400 bp in length, indicating successful integrations of the WY7 and *Hpa1Xoo* into the tobacco plants (Fig 6C). Like the positive control, the WY7-hpa1Xoo transgenic tobacco had significantly higher plant height, fresh weight, dry weight, and chlorophyll content than the *N*. *tabacum* wild type (Fig 7).

#### Soluble proteins produced by WY7-*hpa1Xoo* transgenic tobacco elicited HR on the tobacco leaves

In order to detect whether WY7-*hpa1Xoo* was expressed by the transgenic *N*. *tabacum* and whether that expression resulted in soluble protein production, the proteins from the TI generation of the WY7-*hpa1Xoo* transgenic *N*. *tabacum*, pBI121-*hpa1Xoo* transgenic *N*. *tabacum*, pBI121 transgenic *N*. *tabacum*, and *N*. *tabacum* wild type were extracted.

**Fig 8:**
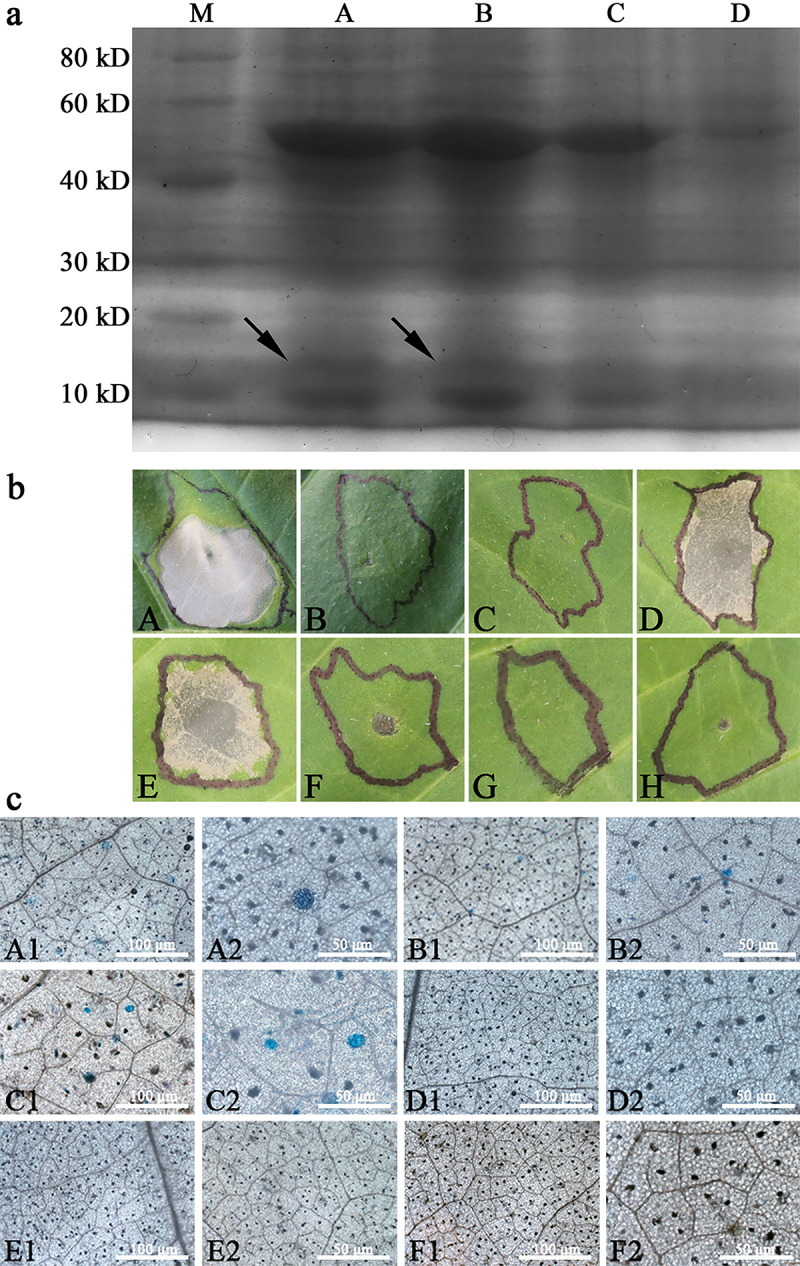
Analysis of soluble proteins from WY7-*hpa1Xoo* transgenic tobacco. **a)** Sodium dodecyl sulfate-polyacrylamide gel electrophoresis (SDS-PAGE) patterns of soluble protein preparations. (A) Soluble protein preparations (1 μL; 301 ng) including harpinXoo from induced T1 generation WY7-*hpa1Xoo* transgenic tobacco. (B) Soluble protein preparations (1 μL; 345 ng) from *N*. *tabacum* wild type; (C) Soluble protein preparations (1 μL; 336 ng) from pBI121-*hpa1Xoo* transgenic plant; (D) Soluble protein preparations (1 μL; 294 ng) including harpinXoo from uninduced T1 generation WY7-*hpa1Xoo* transgenic tobacco. **b)** Soluble proteins of harpinXoo from WY7-*hpa1Xoo* transgenic tobacco could stimulate HR. Bioactivity assays of proteins from leaf tissues, compared with the preparations of GST-harpinXoo from *E*. *coli*. (A) GST-harpinXoo (329 ng/μL) (CK^+^); (B) *N*. *tabacum* wild type (CK^-^) (351 ng/μL); (C) pBI121 transgenic tobacco (351 ng/μL); (D) induced T1 generation WY7-*hpa1Xoo* transgenic tobacco (301 ng/μL); (E) pBI121-*hpa1Xoo* transgenic tobacco (341 ng/μL) (CK^+^); (F) PBS buffer (1 μL); (G) uninduced WY7-*hpa1Xoo* transgenic tobacco (263 ng/μL); (H) ddH_2_O (CK^-^). **c)** Trypan blue staining of WY7-*hpa1Xoo* transgenic tobacco leaves. Micro-HRs were shown as areas stained blue. (A1) and (A2) induced WY7-*hpa1Xoo* transgenic tobacco; (B1) and (B2) pBI121-*hpa1Xoo* transgenic tobacco (CK^+^); (C1) and (C2) with exogenous application of the harpinXoo proteins (CK^+^); (D1) and (D2) pBI121 transgenic tobacco (CK^-^); (E1) and (E2) uninduced WY7-*hpa1Xoo* transgenic tobacco; (F1) and (F2) *N*. *tabacum* wild type (CK^-^).

Through the analysis of sodium dodecyl sulfate-polyacrylamide gel electrophoresis (SDS-PAGE) gel patterns, the harpinXoo protein was determined to have a molecular mass of 15.6 kDa (Fig 8A), which is consistent with that previously reported by Miao [28]. Soluble proteins from transgenic tobacco were injected into the tobacco for HR. HR was induced in WY7-*hpa1Xoo* transgenic tobacco and pBI121-*hpa1Xoo* transgenic tobacco, whereas *N*. *tabacum* wild type and pBI121 transgenic tobacco did not exhibit HR (Fig 8B). As the expression of the exogenous proteins in the transgenic plants is what causes HR, this result suggested that the active harpinXoo protein was expressed in the transgenic *N*. *tabacum*. In addition, after trypan blue staining, it was observed, using light microscopy, that obvious dark-blue micro-HR clusters of necrotic cells were distributed on the leaves of the *N*. *tabacum*. As shown in Fig 8C, the transgenic expression of WY7-*hpa1Xoo* and pBI121-*hpa1Xoo* produced micro-HRs to varying degrees in the transgenic tobacco. The expression of WY7-*hpa1Xoo* produced more micro-HRs than the expression of pBI121-*hpa1Xoo*. The results showed that the transgenic expression of WY7-*hpa1Xoo* produced defensive responses with partial hypersensitive cell death in the transgenic *N*. *tabacum* leaves.

#### TMV resistance

**Fig 9:**
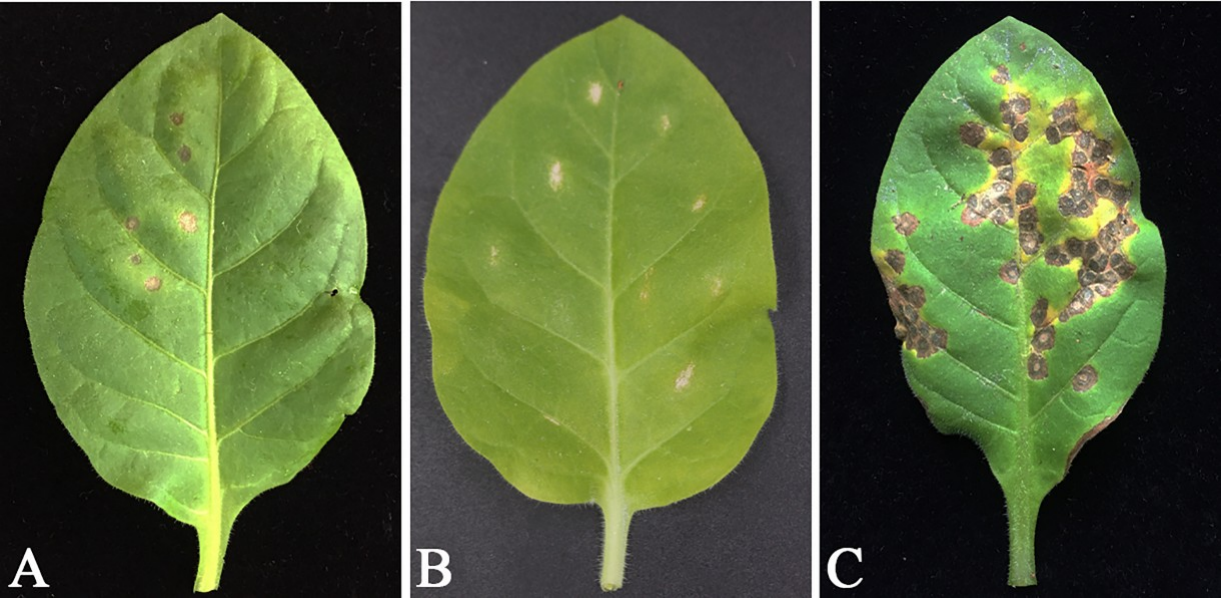
Leaves of transgenic *N*. *tabacum* inoculated with TMV. Leaves transformed with (A) *Hpa1Xoo* or (B) vector pBI121, and (C) *N*. *tabacum* wild type. Lesions are present on the leaves susceptible to TMV. Similar results were obtained from five sets of experiments: a total of 30 plants of each genotype were inoculated.

The T1 generation of the WY7-*hpa1Xoo* transgenic tobacco was induced (low-temperature induction) and then inoculated with TMV. The *hpa1Xoo* transgenic tobacco had significantly higher TMV resistance than the *N*. *tabacum* wild type (Fig 9). The number of lesions observed on the *N*. *tabacum* wild type after inoculation with TMV was significantly higher than that of the positive control 35S-*hpa1Xoo* and WY7-*hpa1Xoo* transgenic T1 plants (*P* < 0.01; Table 3). Compared with the *N*. *tabacum* wild type, the average number of lesions observed on the positive control group was 45.91% lower. Compared with the *N*. *tabacum* wild type and the positive control, the average number of lesions observed on the WY7-*hpa1Xoo* transgenic T1 plants were 61.47% and 28.77% lower, respectively.

**Table 3:** Resistance levels of WY7-*hpa1Xoo* transgenic tobacco against TMV.

Plants	Plant leaf lesion number (mean±SE)	*P* ≤ 0.01 Significance level^a^	Lesion reduction (%)
*N*. *tabacum* wild type	40.67±4.93	A	
T1 generation of 35S*-hpa1Xoo* transgenic tobacco	22.00±3.60	B	45.91
T1 generation of WY7-*hpa1Xoo* transgenic tobacco	15.67±3.51	B	61.47

SE, standard error.

^a^ *P* ≤ 0.01 Significance level, Different letters indicate statistically significant differences between treatments according to Duncan's multiple range test at *P* ≤ 0.01.

## Discussion

In this study, we identified and developed an effective endogenous promoter of the obligate biotrophic fungus *O*. *heveae* that may be a useful tool for genetic engineering.

In recent years, there have been several reports of exogenous gene expression regulation by filamentous fungal promoters in non-hosts. Most of these investigations used endogenous promoters of the host to drive the expression of the endogenous genes. Yin *et al*. discovered a pH-inducible promoter *Pgas* from *Aspergillus niger*, which can initiate the expression of the aconitate decarboxylase gene, giving *A*. *niger* the ability to synthesize itaconic acid [29]. Antunes *et al*. found a benzoic acid-induced *bphA* promoter in *A*. *niger* [30]. Meanwhile, Blatzer *et al*. found the inducible promoter *xyl1*^*P*^ was induced by xylose and xylan in *Acremonium chrysogenum* [31]. Zambanini *et al*. reported two inducible promoters, *P*_*tad1*_ and *P*_*mtt1*_, that were from the itaconic acid gene cluster of *Ustilago maydis* Corola; the induction conditions for these two promoters were found to be limited by nitrogen [32]. If promoters such as 35S can regulate the expression of exogenous genes in non-hosts, even across kingdoms, they will certainly prove highly beneficial in transgenic engineering and enable the successful expression of diverse exogenous genes.

Obligate biotrophic fungi cannot be cultured *in vitro* owing to their obligate parasitic characteristics, making operations such as genetic transformation very difficult. Therefore, research on this organism class is very limited, especially in molecular biology. To date, very few endogenous promoters of obligate biotrophic fungi have been reported. For the obligate parasitic fungus *O*. *heveae*, most previous research has focused on its physiology, rather than on its molecular biology; thus, almost no endogenous promoters have been reported for this species. WY7 is derived from *O*. *heveae*. In this investigation, we have found that the expression of WY7 can regulate endogenous gene expression in both monocots and dicots.

Few promoters can regulate highly efficient gene expression in both dicots and monocots, including the 35S and ACT1 promoters. Liu *et al*. reported that the *GUS* gene was regulated by the 35S promoter and Ubi1 promoter in the callus of *Populus simonii*; moreover, the expression activity of the 35S promoter was 3.7 times higher than that of the Ubi1 promoter [33]. Wang and Oard reported that the ability of the *RUBQ2* promoter to drive *GUS* gene expression was higher than that of the 35S promoter by 28 and 35 times in the tillering and heading stages, respectively [34]. Schledzewski and Mendel reported that the expression of the *GUS* gene under the Ubiquitin1 promoter and the ACT1 promoter in maize was 13.2 and 11.1 times higher than that under the 35S promoter, respectively, whereas, in barley, it was 11.9 and 6.4 times higher, respectively [35]. In our study, the expression activity of the WY7 promoter was 11.7 times higher than that of the 35S promoter in *N*. *tabacum*. In *O*. *sativa*, the expression activity of the WY7 promoter was 5.13 times higher than that of the ACT1 promoter. We found that, in monocots and dicots, the WY7 promoter is much more active than the ACT1 promoter and the 35S promoter, respectively. These results indicate that the WY7 promoter could have a large range of applications in both monocots and dicots.

Promoters can be classified into constitutive promoters, inducible promoters, and tissue/organ-specific promoters, according to their different transcriptional regulations [36]. Most early promoter studies focused on constitutive promoters. With research on transcriptional regulation and gene expression intensifying, promoter studies in recent years have increasingly focused on inducible and tissue/organ-specific promoters. Constitutive promoters have been widely used in the field of plant genetic engineering, including the ACT1 promoter, derived from rice, and the 35S promoter, derived from the cauliflower mosaic virus (CaMV) [37, 38]. However, constitutive promoters have some disadvantages, as they drive the expression of exogenous genes in all tissues and organs and can have some negative effects owing to their lack of temporal and spatial specificity. For example, gene overexpression in plants can cause disease symptoms, gene silencing, and the wasting of energy and nutrients [39, 40]; further, the excessive accumulation of gene products causes metabolic disorders, which affect plant growth and, in severe cases, may cause death [41]. Tissue/organ-specific promoters can initiate the expression of genes during plant development or in specific tissues and organs, avoiding the overexpression of exogenous genes and saving energy for the plant [42]. An inducible promoter can efficiently induce the transcriptional expression of a gene under specific conditions, compared to a tissue/organ-specific promoter. Only upon receiving the stimulation signal does the inducible promoter regulate the downstream gene for efficient expression or more effectively control the exogenous genes [43]. Consequently, it is no surprise that, in recent years, the focus of promoter research has gradually shifted from constitutive promoters toward inducible and tissue/organ-specific promoters.

The constitutive promoter has no spatio-temporal specificity, but the inducible promoter can induce the expression product from the target gene in specific time periods, and in response to physical and chemical signals [44–48]. There are three unnamed cis-acting elements in the WY7 fragment. Our experimental analysis of common types of induction, including high temperatures, low temperatures, drought stress, salt stress, and light, showed that WY7 was a low temperature- and salt stress-inducible promoter.

As an elicitor, the harpin protein can induce plants to produce a variety of beneficial phenotypes, such as disease resistance and enhanced growth. Strobel *et al*. reported that HrpZPss induced systemic acquired resistance in cucumber in response to a diverse array of pathogens [49]. Dong *et al*. reported that harpins not only induced insect resistance in *Arabidopsis thaliana* but also promoted the growth of the plant shoots and roots [50]. Another study showed that the Hpal110-42 fragment was 1.3 to 7.5 times more effective than the full length, and demonstrated that external application of Hpal110-42 to *Impatiens balsamina* L. can promote flower growth and retard senescence of the fully expanded flowers [51]. Anil and Podile reported that the elicitor protein harpin_Pss_ promoted the growth of groundnut [52]. Finally, Xu *et al*. reported that the *hpaG*_*Xoo*_ transgenic chrysanthemum flowered earlier than the chrysanthemum wild type, concluding that the *hpaG*_*Xoo*_ gene likely promoted chrysanthemum development [53]. Similarly, by measuring the phenotype of WY7-*hpa1Xoo* transgenic tobacco, we found that harpinXoo promoted growth in *N*. *tabacum*, like the other harpin proteins.

HR is considered to be the active response of plants to pathogens [54]. Plants exhibit programmed cell death (PCD) during HR in response to certain pathogens [55]. Previous investigations have shown that hpaXm was able to induce HR in plants [56]. However, Peng *et al*. found that, although *HpalXoo* transgenic tobacco could induce HR, it was unable to cause PCD [27]. Wei *et al*. reported that HR was elicited by harpin_Ea_ in *Erwinia amylovora* [4]. Similar to previous investigations, we found that *Hpa1Xoo* can express active proteins in transgenic *N*. *tabacum*. There was no visible macro-HR on WY7-*hpa1Xoo* transgenic *N*. *tabacum* leaves, but micro-HR dead cell clusters were observed after trypan blue staining, and the TMV resistance of transgenic *N*. *tabacum* was enhanced. Our results showed that *HpalXoo* can be expressed under the regulation of WY7.

Our study fills an important gap in the scientific knowledge of *O*. *heveae* molecular biology. Since the promoter is an important regulatory sequence, the in-depth study of the *O*. *heveae* promoter will improve our understanding of its transcriptional regulation mechanisms and facilitate the establishment of a genetic transformation system for *O*. *heveae*. The wide range of exogenous gene expression regulated by the WY7 promoter also indicates that WY7 has the potential to be a useful and widely applicable tool in genetic engineering.

## Supporting information

S1 Fig The original image of Fig 1A. WY7 Amplification from the genome of *O*. *heveae*. (M) Marker 2000; (A1) and (A2) WY7; (CK^-^) Negative control with ddH_2_O as template.
(DOCX)

S2 Fig The original image of Fig 4B. PCR verification of WY7 in transgenic tobacco. (M) Marker 2000; (CK^-^) Negative control with ddH_2_O as template; (A) WY7.
(DOCX)

S3 Fig The original image of Fig 4C. PCR-Southern blot analysis of transferred DNA sequences in the genomes of WY7-*GUS* transgenic tobacco obtained after *Agrobacterium tumefaciens*-mediated transformation (ATMT). (M) DNA molecular weight marker (DIG-labeled); (A) WY7; (CK^–^) *N*. *tabacum* wild type. The unlabeled lanes are other samples not related to this study.
(DOCX)

S4 Fig The original image of Fig 6B. PCR verification of WY7-*Hpa1Xoo* transgenic tobacco plants. (M) Marker 2000; (A) WY7-*Hpa1Xoo* transgenic tobacco plants, primers: WY7F/R; (B) WY7-*Hpa1Xoo* transgenic tobacco plants, primers: Hpa1XooF/R; (C) CK^+^, template: *O*. *heveae* wild type, primers: WY7F/R; (D) CK^+^, template: *X*. *oryzae* wild type, primers: Hpa1XooF/R; (E) CK^+^, template: recombinant vector pBI121-WY7-*Hpa1Xoo*, primers: WY7F/R; (F) CK^+^, template: recombinant vector pBI121-WY7-*Hpa1Xoo*, primers: Hpa1XooF/R; (G) CK^+^, template: 35S-*Hpa1Xoo* transgenic tobacco plants, primers: 35SF/R; (H) CK^+^, template: 35S-*Hpa1Xoo* transgenic tobacco plants, primers: Hpa1XooF/R; (I) CK^-^, template: *N*. *tabacum* wild type, primers: WY7F/R; (J) CK^-^, template: *N*. *tabacum* wild type, primers: Hpa1XooF/R; (K) CK^-^, template: ddH_2_O, primers: WY7F/R; (L) CK^-^, template: ddH_2_O, primers: Hpa1XooF/R.
(DOCX)

S5 Fig The original image of Fig 6C. PCR-Southern blot analysis of transferred DNA sequences in the genomes of WY7-*Hpa1Xoo* transgenic tobacco obtained after *Agrobacterium tumefaciens*-mediated transformation (ATMT). (M) DNA molecular weight marker (DIG-labeled); (CK^–^) *N*. *tabacum* wild type; (A) WY7; (B) *Hpa1Xoo*. The unlabeled lanes are other samples not related to this study.
(DOCX)
