# Supplementary material for: WY7 is a newly identified promoter from the rubber powdery mildew pathogen that regulates exogenous gene expression in both monocots and dicots
Source: PLoS One. 2020 Jun 1;15(6):e0233911. doi: 10.1371/journal.pone.0233911 (PMC7263610; doi:10.1371/journal.pone.0233911)
Supplement: S1 Fig — WY7 Amplification from the genome of O. heveae. (M) Marker 2000; (A1) and (A2) WY7; (CK-) Negative control with ddH2O as template. (DOCX) [file pone.0233911.s001.docx]

**

**

**Fig S1**
