# Supplementary figures and images for: WY7 is a newly identified promoter from the rubber powdery mildew pathogen that regulates exogenous gene expression in both monocots and dicots
Source: PLoS One. 2020 Jun 1;15(6):e0233911. doi: 10.1371/journal.pone.0233911 (PMC7263610; doi:10.1371/journal.pone.0233911)

**
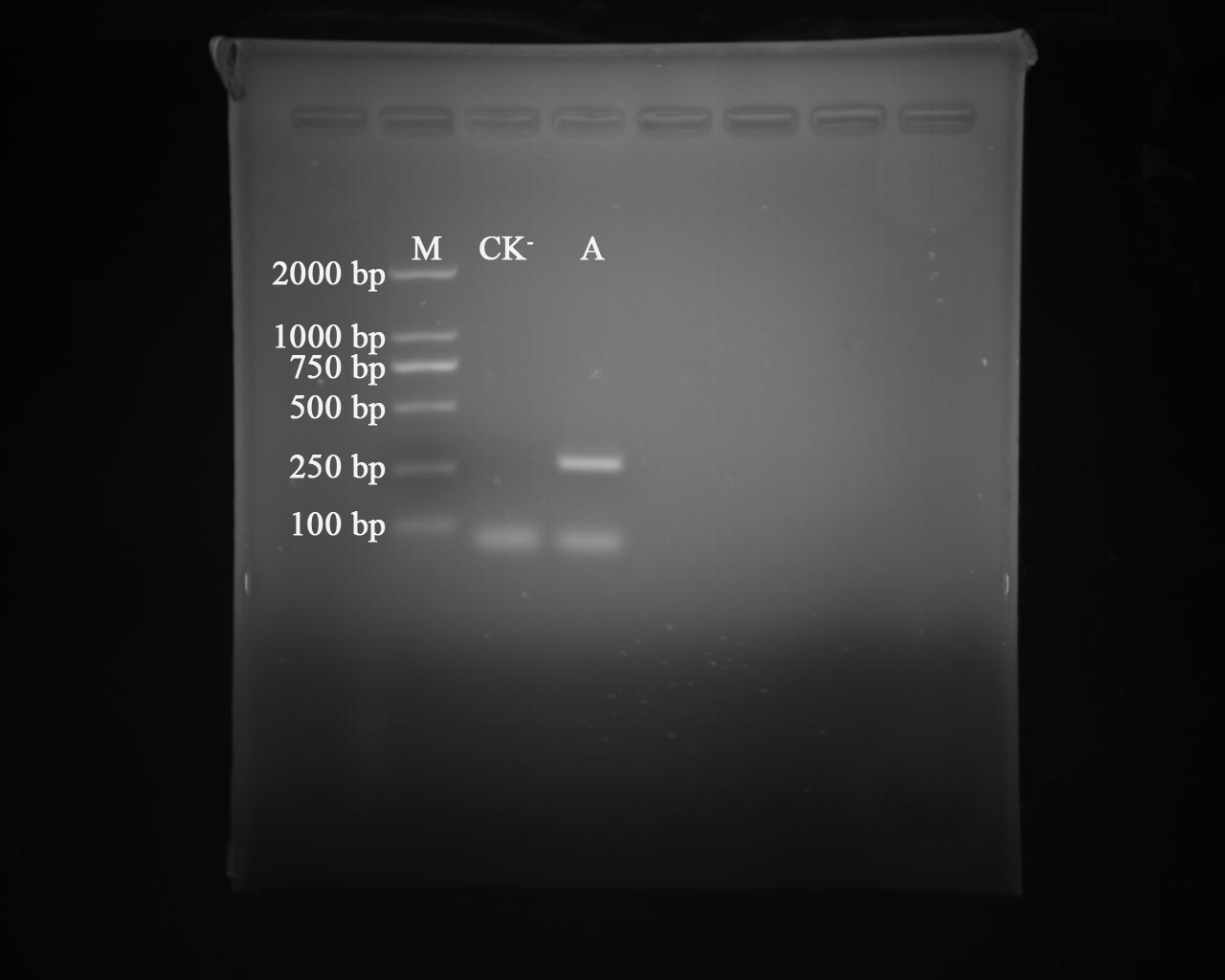
**

**Fig S2**

Supplement: S2 Fig — PCR verification of WY7 in transgenic tobacco. (M) Marker 2000; (CK-) Negative control with ddH2O as template; (A) WY7. (DOCX) [file pone.0233911.s002.docx]

**
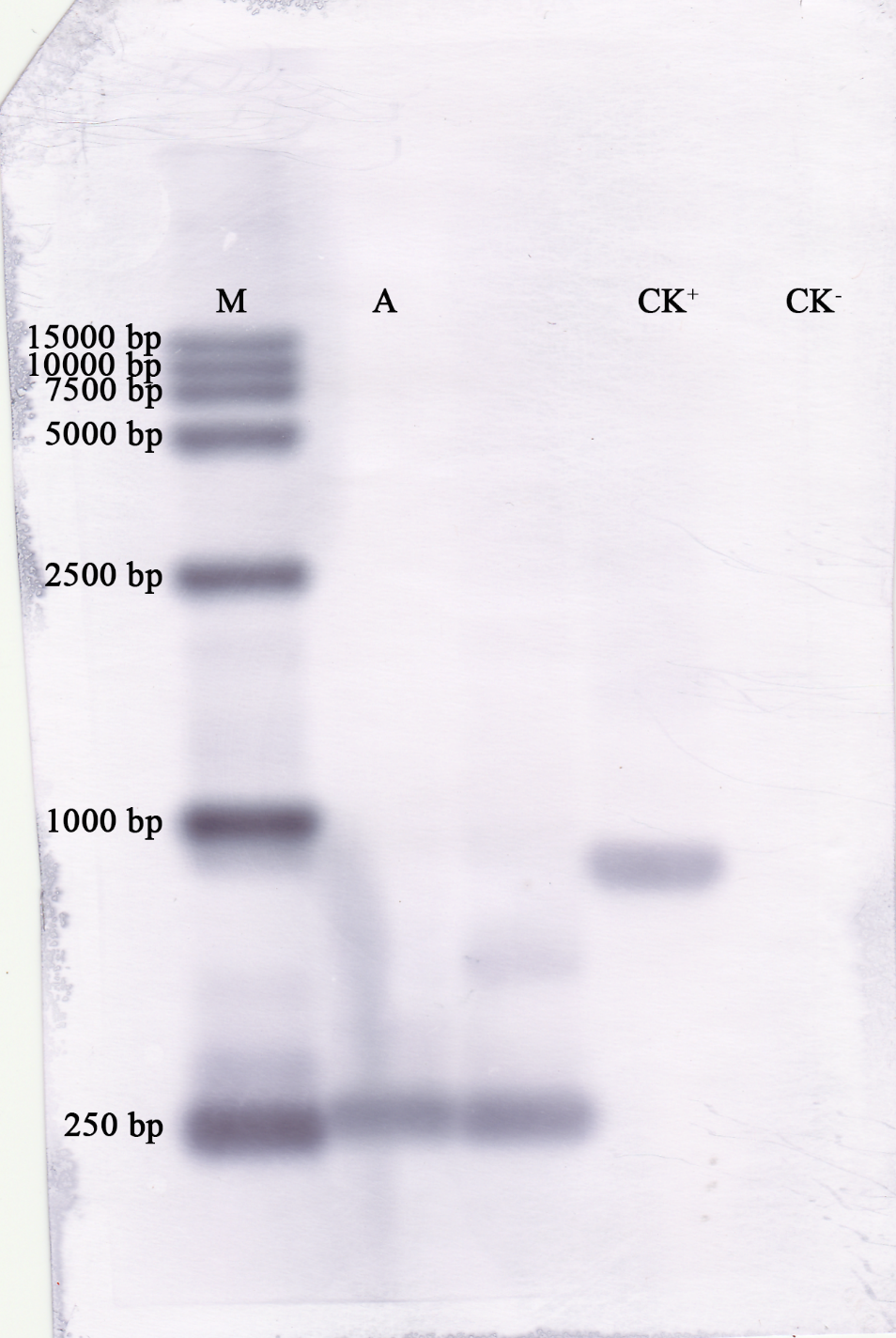
**

**Fig S3**

Supplement: S3 Fig — PCR-Southern blot analysis of transferred DNA sequences in the genomes of WY7-GUS transgenic tobacco obtained after Agrobacterium tumefaciens-mediated transformation (ATMT). (M) DNA molecular weight marker (DIG-labeled); (A) WY7; (CK–) N. tabacum wild type. The unlabeled lanes are other samples not related to this study. (DOCX) [file pone.0233911.s003.docx]

**
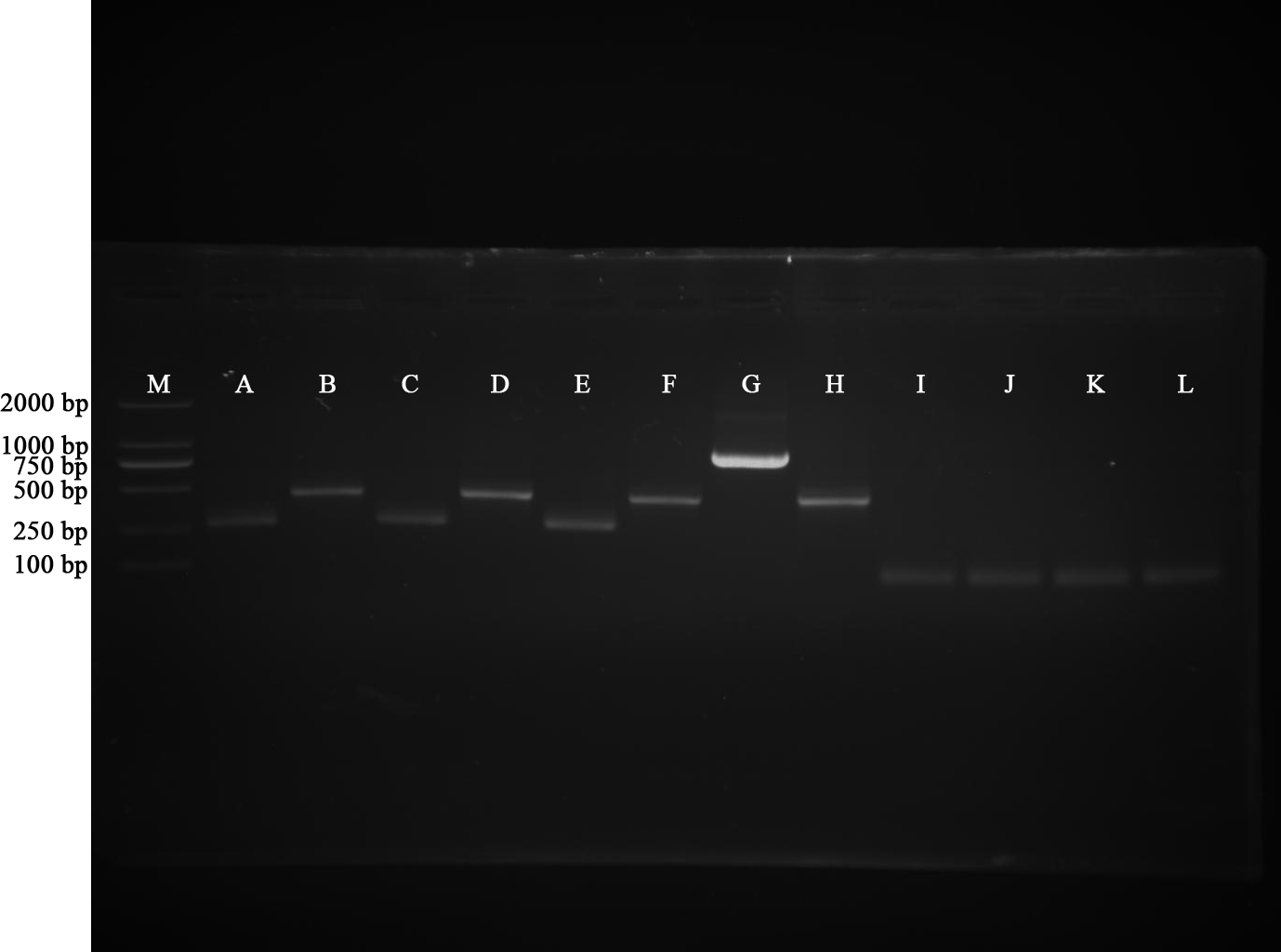
**

**Fig S4**

Supplement: S4 Fig — PCR verification of WY7-Hpa1Xoo transgenic tobacco plants. (M) Marker 2000; (A) WY7-Hpa1Xoo transgenic tobacco plants, primers: WY7F/R; (B) WY7-Hpa1Xoo transgenic tobacco plants, primers: Hpa1XooF/R; (C) CK+, template: O. heveae wild type, primers: WY7F/R; (D) CK+, template: X. oryzae wild type, primers: Hpa1XooF/R; (E) CK+, template: recombinant vector pBI121-WY7-Hpa1Xoo, primers: WY7F/R; (F) CK+, template: recombinant vector pBI121-WY7-Hpa1Xoo, primers: Hpa1XooF/R; (G) CK+, template: 35S-Hpa1Xoo transgenic tobacco plants, primers: 35SF/R; (H) CK+, template: 35S-Hpa1Xoo transgenic tobacco plants, primers: Hpa1XooF/R; (I) CK-, template: N. tabacum wild type, primers: WY7F/R; (J) CK-, template: N. tabacum wild type, primers: Hpa1XooF/R; (K) CK-, template: ddH2O, primers: WY7F/R; (L) CK-, template: ddH2O, primers: Hpa1XooF/R. (DOCX) [file pone.0233911.s004.docx]

**
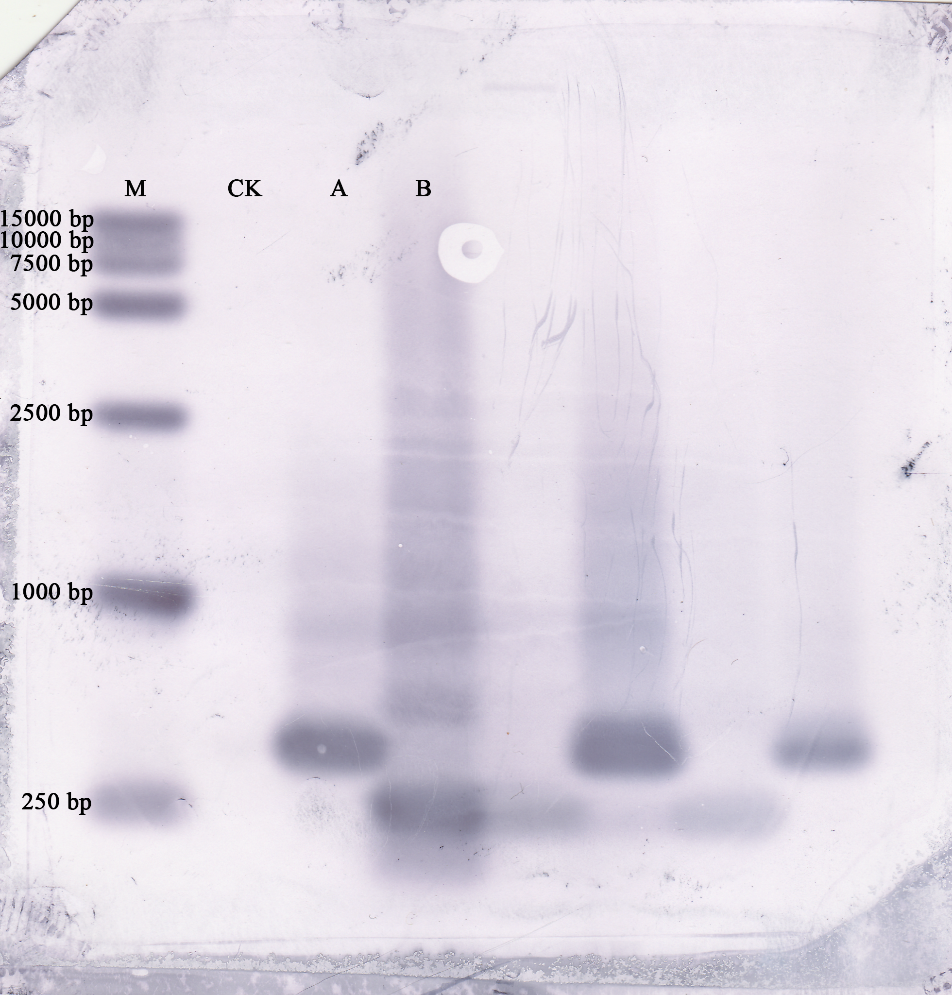
**

**Fig S5**

Supplement: S5 Fig — PCR-Southern blot analysis of transferred DNA sequences in the genomes of WY7-Hpa1Xoo transgenic tobacco obtained after Agrobacterium tumefaciens-mediated transformation (ATMT). (M) DNA molecular weight marker (DIG-labeled); (CK–) N. tabacum wild type; (A) WY7; (B) Hpa1Xoo. The unlabeled lanes are other samples not related to this study. (DOCX) [file pone.0233911.s005.docx]
